# miR-192-5p suppresses the progression of lung cancer bone metastasis by targeting TRIM44

**DOI:** 10.1038/s41598-019-56018-5

**Published:** 2019-12-23

**Authors:** Peng Zou, Menghai Zhu, Chong Lian, Jiaqiang Wang, Zhiquan Chen, Xiaoming Zhang, Yongchao Yang, Xinfeng Chen, Xinhui Cui, Jijun Liu, Hexuan Wang, Qi Wen, Ji Yi

**Affiliations:** 1 Department of orthopedics, Zhengzhou Seventh People’s Hospital, Zhengzhou, China; 2 The First Affiliated Hospital of Sun Yat-sen University, GuangZhou, China; 3 Laboratory of Immunotherapy, The Affiliated Cancer Hospital of Zhengzhou University, Henan Cancer Hospital, Zhengzhou, China; 4 Henan Provincial People’s Hospital, Zhengzhou University, Zhengzhou, China

**Keywords:** Bone metastases, RNAi

## Abstract

Lung cancer is the leading cause of cancer-related deaths worldwide, with 50–70% of patients suffering from bone metastasis. Accumulating evidence has demonstrated that miRNAs are involved in cell proliferation, migration, and invasion in malignancy, such as lung cancer bone metastasis. In the present study, we demonstrated that reduced miR-192-5p and increased TRIM44 levels were associated with the proliferation, migration and invasion of lung cancer. Furthermore, the potential functions of miR-192-5p were explored in A549 and NCI-H1299 cells. We found that miR-192-5p upregulation suppressed tumour behaviours in lung cancer cells. To further investigate whether miR-192-5p is associated with TRIM44, we used TargetScan software to predict the binding site between miR-192-5p and TRIM44. Luciferase activity assays were performed to verify this prediction. In addition, the significant role of miR-192-5p in negatively regulating TRIM44 expression was manifested by our research group. our results suggest that miR-192-5p inhibited the proliferation, migration and invasion of lung cancer through TRIM44.

## Introduction

Lung cancer is the leading cause of cancer-related deaths worldwide, with 50–70% of patients suffering from bone metastasis^1–5^. Detrimental skeletal-related events are more common in end-stage patients and are associated with significant morbidity^6–8^. Because of the unsatisfactory outcome of bone metastasis^9,10^, further research should be performed to explore the underlying mechanisms involved in lung cancer proliferation, invasion, and metastasis.

MicroRNAs (miRNAs/miRs) are endogenous, small, noncoding and highly conserved RNAs^11,12^. Recent studies have revealed that miRNAs are implicated in various cellular processes^13–15^. In addition, many studies have already demonstrated that miRNA dysfunction is involved in the occurrence and progression of malignancies, such as breast cancer, gastric carcinoma, prostate cancer, and lung cancer^16–19^. In recent years, various studies have shown that miR-192-5p is significantly downregulated in malignant carcinoma, while miR-192-5p overexpression may play a potential role in suppressing tumourigenesis through different mechanisms^20,21^. In lung cancer bone metastasis, miR-192-5p plays a major role in inhibiting tumourigenesis^22,23^. However, the underlying mechanisms of how miR-192-5p is involved in the development and progression of lung cancer bone metastasis remain largely unknown.

Tripartite motif 44 (TRIM44) is a TRIM family protein that is involved in the ubiquitination and degradation of target proteins by modulating E3 ubiquitin ligases. It has been reported that TRIM44 is overexpressed in various pathological conditions, including developmental disorders, as well as in cell cycle regulation and DNA damage responses^24^. Previous reports have revealed that TRIM functions as a marker for the degradation of target proteins through the proteasome, but it also regulates protein-protein interactions and enzyme activation^25,26^. Recently, increasing evidence suggests that TRIM44 is involved in oncogenesis and tumourigenesis in human malignant cancers^27,28^. However, the biological function of TRIM44 in lung cancer bone metastasis remains unclear.

As such, we propose that miR-192-5p upregulation in patient serum and lung cancer cell lines inhibits lung cancer metastasis, possibly by downregulating TRIM44.

## Results

### miR-192-5P expression in the serum of lung cancer patients and in lung cancer cell lines

**Figure 1:**
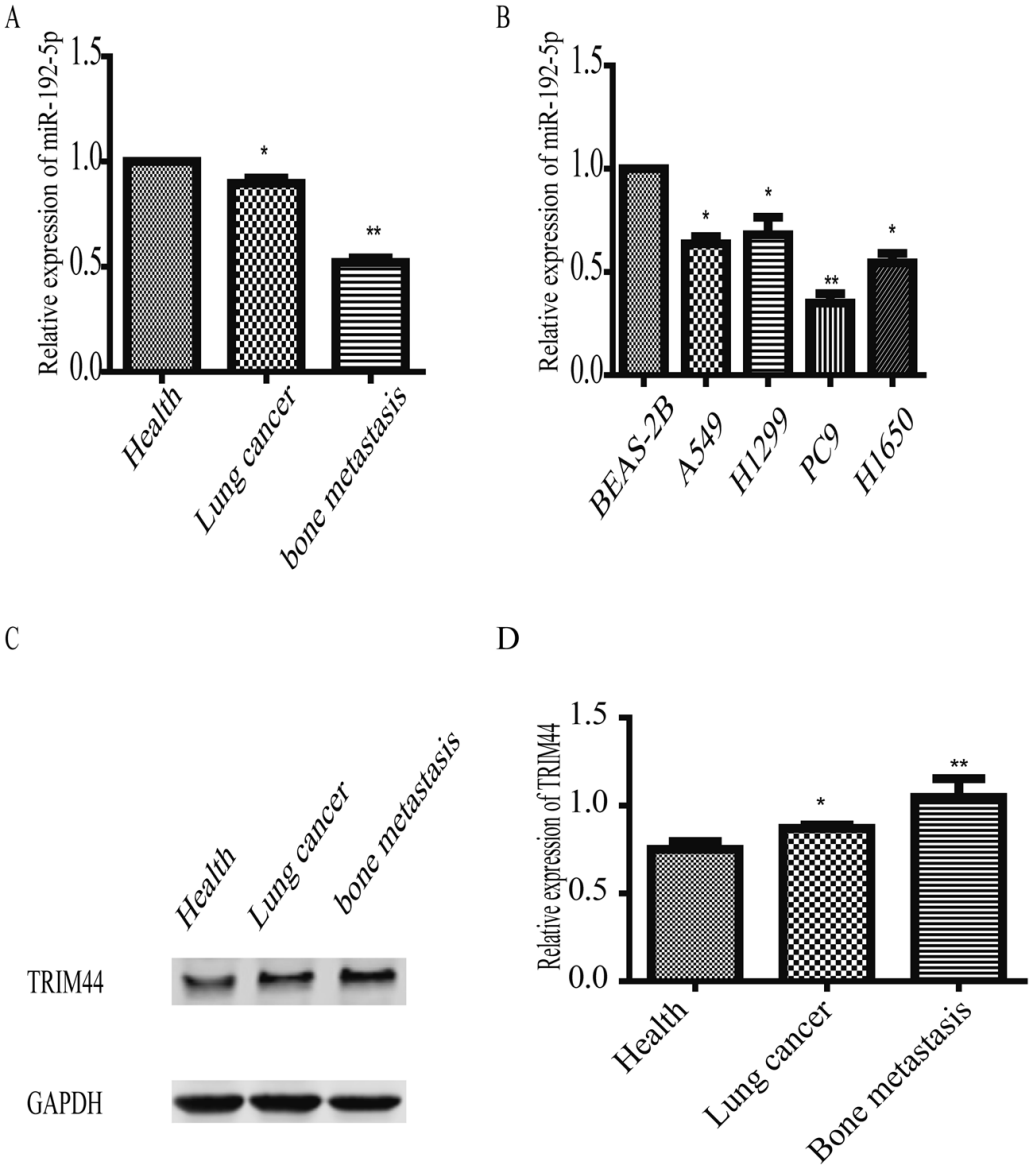
Downregulation of miR-192-5P in human patient serum and lung cancer cell lines. (**A**) miR-192-5P expression in healthy volunteers and in patients with and without lung cancer bone metastasis. GAPDH mRNA was used as an internal control. (**B**) miR-192-5P expression was significantly lower in human lung cancer cells than in normal lung epithelial cells. GAPDH was used as an internal control. (**C**) TRIM44 expression was the highest in three tissue groups. GAPDH was used as an internal control. (**D**) Quantification of TRIM44 was normalized to GAPDH. Each bar represents the mean ± SEM. *P < 0.05, **P < 0.01, NS, non-significant. This study investigated serum from healthy volunteers (n = 130), lung cancer patients without bone metastasis (n = 68) and lung cancer patients with bone metastasis (n = 78).

miR-192-5p expression was measured in serum samples from healthy volunteers (n = 130), lung cancer patients without bone metastasis (n = 68) and lung cancer patients with bone metastasis (n = 78). As shown in Fig. 1A, qPCR analysis indicated that the abundance of miR-192-5p was significantly lower in lung cancer patients with bone metastasis than in healthy volunteers and lung cancer patients without bone metastasis. Moreover, NCI-H1299, H1650, PC9, A549, BEAS-2B cells were cultured. miR-192-5p levels were dramatically lower in lung cancer cells than in BEAS-2B cells (Fig. 1B). In addition, our data revealed that TRIM44 mRNA expression was significantly upregulated in lung cancer cells compared with that in BEAS-2B cells (Fig. 1C), which was consistent with the western blot analysis (Fig. 1D). Clinical characteristics of the patients are summarized in Table 1. These results suggested that miR-192-5p expression was lower in lung cancer patients with bone metastasis than in healthy volunteers and lung cancer patients without bone metastasis.

**Table 1:** Clinical and sociodemographic characteristics of the population studied.

Clinicopathological characteristics	Healthy volunteers(Num)	Lung cancer without bone metastasis(Num)	Lung cancer with bone metastasis(Num)
**Ages**			
<65	60	21	17
≥65	50	48	61
**Gender**			
Male	62	32	36
Female	68	37	42
**TNM stage**			
I-II		69	
III-IV			78
**Lymph node metastasis**			
Positive		58	69
negative		11	9
**Tumor Size**			
≤4 cm		27	30
>4 cm		41	48
**Differentiation**			
Well		36	31
Poor		33	47

### Ectopic expression of miR-192-5p affects the proliferation, migration and invasion of lung cancer cells

**Figure 2:**
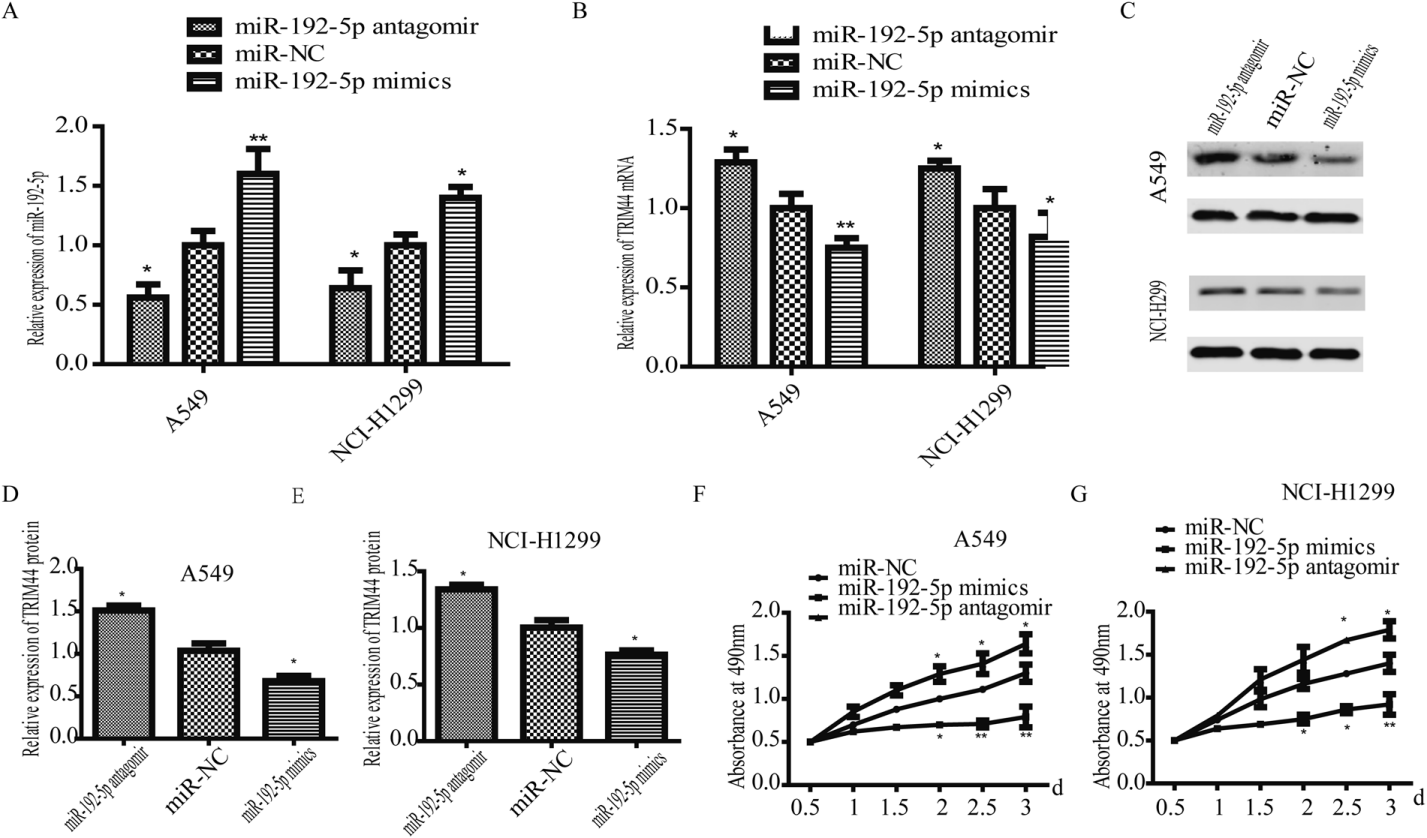
miR-192-5p upregulation suppressed lung cancer cell growth. (**A**) A549 and NCI-H1299 cells were transfected with miR-192-5p antagomir, miR-NC, and miR-192-5P mimic. High miR-192-5p expression was examined by qPCR assay. (**B**) TRIM44 downregulation was detected by qPCR (**B**) and western blotting (**C**). Quantification of TRIM44 was normalized to GAPDH (**D**,**E**). MTT assays suggest that cell viability was enhanced upon miR-192-5p downregulation in (**F**) A549 and (**G**) NCI-H1299 cells. *P < 0.05, **P < 0.01, ***P < 0.001. All experiments were performed at least three times.

**Figure 3:**
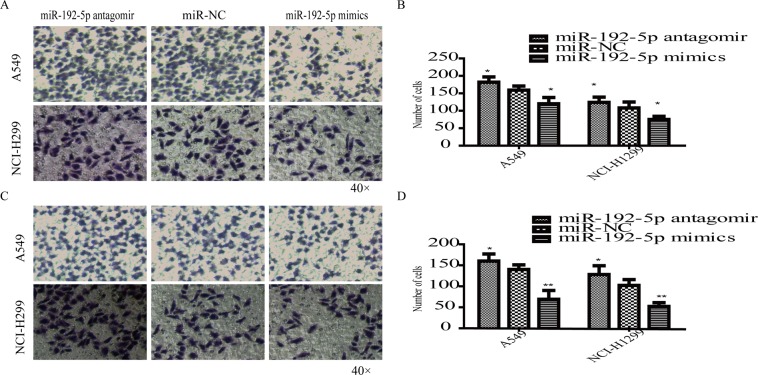
miR-192-5P upregulation suppressed cell migration and invasion. (**A**) A549 and NCI-H1299 cells transfected with miR-192-5p antagomir, miR-NC, and miR-192-5P mimic were subjected to cell migration assays, and the cells were stained with a crystal violet solution. (**B**) The number of migrated cells was reduced by miR-192-5p. (**C**) Images of the invasion assays are shown, and (**D**) the invasive ability was weakened. NC, non-treated control group. Each bar represents the mean ± SEM. *P < 0.05, **P < 0.01. All experiments were performed at least three times with duplication.

miR-192-5p expression was regulated by transfecting a miR-192-5p antagomir and miR-192-5p mimics into A549 and NCI-H1299 cells. miR-192-5p expression was verified by RT-qPCR analysis (Fig. 2A), and mRNA (Fig. 2B) and protein (Fig. 2C–E) levels of TRIM44 were detected by RT-qPCR and western blotting. As shown in Fig. 2A–D, miR-192-5p expression was significantly downregulated in lung cancer cells. Furthermore, MTT assays revealed that the proliferation of both A549 and NCI-H1299 cells was promoted due to miR-192-5p downregulation (Fig. 2F,G). The migration and invasion assays showed that a increase in miR-192-5p significantly suppressed cell migration and invasion (Fig. 3A–D). These data revealed that miR-192-5p inhibited the growth, migration and invasion of lung cancer cells.

### TRIM44 was the direct target of miR-192-5p in lung cancer cells

**Figure 4:**
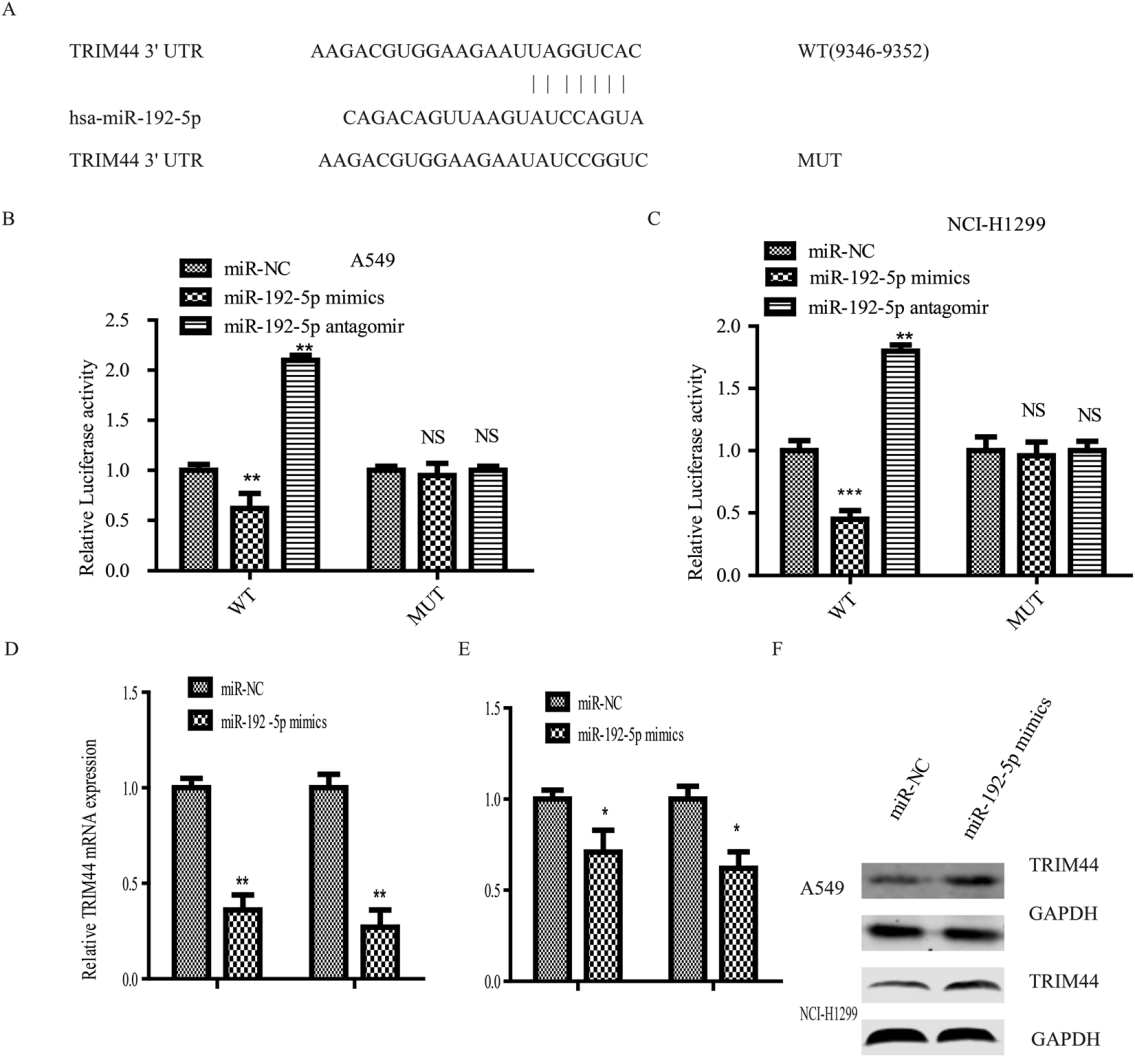
TRIM44 is a direct target of miR-192-5p. (**A**) Potential targets of miR-192-5p were located in the 3′-UTR of TRIM44 through TargetScan Human. (**B**) A549 and (**C**) NCI-H1299 cells transfected with both miR-192-5p mimic and miR-192-5P antagomir in the presence of wild-type or mutant TRIM44 3′-UTR was detected though luciferase activity. (**D**) qPCR and western blotting (**E**) were performed to identify the mRNA and protein expression levels of TRIM44 in transfected cells. Quantification of TRIM44 was normalized to GAPDH. All experiments were performed at least three times with duplication within each individual experiment.

We used TargetScan and miRNA databases to predict the downstream targets of miR-192-5p and further explore the underlying molecular mechanism involved in the growth of lung cancer cells. The TRIM44 3′-UTR and miR-192-5p sequences were predicted to bind to one another (Fig. 2A). Thus, to further explore whether TRIM44 is directly targeted by miRNA-192-5p, a luciferase reporter assay was established, and miR-192-5p transfection significantly decreased the luciferase activity of the wild-type, but not the mutant, TRIM44 3′-UTR (Fig. 4B). Conversely, a decrease in miR-192-5p significantly contributed to the activity of wild-type TRIM44 in A549 and NCI-H1299 cells (Fig. 4C). Furthermore, the data revealed that miR-192-5p overexpression dramatically decreased the mRNA level of TRIM44 (Fig. 4D). According to the western blot analysis, high miR-192-5p expression significantly decreased the protein abundance of TRIM44 in both A549 and NCI-H1299 cells (Fig. 4E,F).

### miR-192-5p regulated the proliferation and metastatic behaviours of lung cancer cells through TRIM44

**Figure 5:**
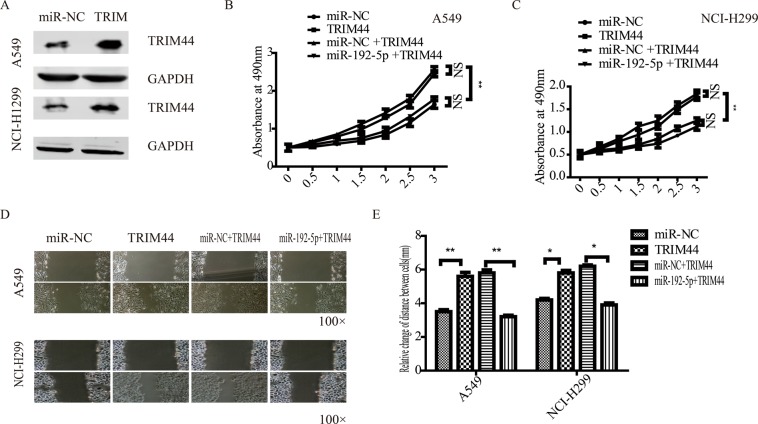
miR-192-5p negatively regulated TRIM44 expression in lung cancer cells. (**A**) A549 and NCI-H1299 cells were transfected with TRIM44 or plasmid vectors. A549 (**B**) and NCI-H1299 (**C**) cell viability was determined by MTT assay. (**D**) Representative images of A549 and NCI-H1299 cells showing cell migration various times after wounding. More cells migrated into the wound at 36 h in TRIM44 and miR-NC + TRIM44 cells than in miR-NC and miR-192-5p + TRIM44 cells. All experiments were performed at least three times.

To verify the effects of miR-192-5p on proliferation, lung cancer cell metastatic behaviour was assessed after directly targeting TRIM44. A549 and NCI-H1299 cells expressing miR-192-5p or miR-NC were transfected with Flag-vector or Flag-TRIM44. According to the western blot assay, TRIM44 protein expression increased in A549 and NCI-H1299 cells (Fig. 5A). MTT assays (Fig. 5B,C) demonstrated that the proliferation of both A549 and NCI-H1299 cells was promoted by TRIM44, and miR-192-5p reversed this effect. Consistent with these data, miR-192-5p significantly attenuated the promotion effects of TRIM44 on lung cancer cell migration. These results indicated that miR-192-5p may suppress lung cancer cells by targeting TRIM44.

### The potential role of miR-192-5P in inhibiting tumour growth ***in vivo***

**Figure 6:**
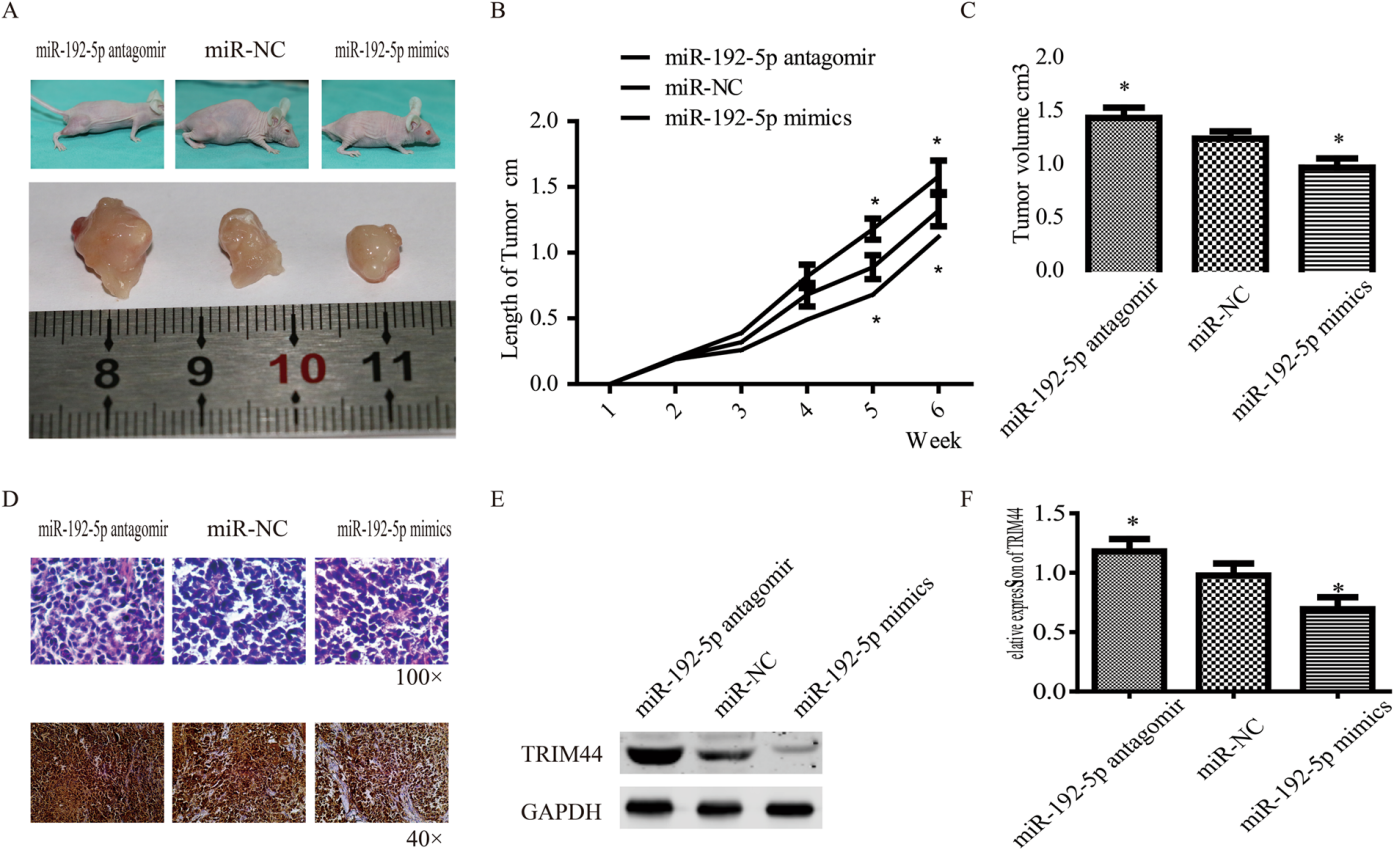
Effect of miR-192-5P on tumour growth *in vivo*. A lung cancer bone metastasis model was established by injecting A549 cells (5 × 10^6^ cells) into the marrow cavity of nude mice (12 mice per group). (**A**) Representative images of femur tumours in nude mice. (**B**) Tumour size was measured using Vernier callipers once a week until the animals were sacrificed. (**C**) Tumour volume was measured at the last time point. (**D**) Representative H&E staining (100× magnification) and immunohistochemistry images for TRIM44 (40× magnification) in tumour sections from different groups. TRIM44 protein expression in tumour tissues was examined by western blotting (**E**) and quantified (**F**). Each bar represents the mean ± SEM. *P < 0.05.

To further verify our observation, *in vivo* experiments were carried out by injecting A549 cells (5 × 10^6^ cells) into the bone marrow of BALB/c-nu/nu mice. One week later, the mice were subjected to injections (30 mg/kg) of the chimeric anti-miRs to suppress miR-192-5p (miR-192-5p antagomir) and promote miR-192-5p (miR-192-5p mimics); miR-NC was used as a control. Treatment with miR-192-5p markedly decreased the tumour sizes (Fig. 6A–C). Then, the tumours were examined by H&E and immunohistochemical staining to determine TRIM44 protein expression; the miR-192-5p mimic group demonstrated obviously lower expression (Fig. 6D). Finally, western blot analyses of tumour tissues showed that miR-192-5p decreased TRIM44 protein expression (Fig. 6E,F). These data suggested that overexpression of miR-192-5p suppressed tumour growth *in vivo*.

## Discussion

Accumulating evidence has demonstrated that miRNAs are involved in cell proliferation, migration, and invasion in malignancy, such as lung cancer bone metastasis^11,13,14,17,19^. For instance, HE *et al*.^29^ revealed that downregulation of miR-20b may dramatically suppress H22 cell proliferation by directly targeting *PTEN*. In the present study, we demonstrated that miR-192-5p reduction and TRIM44 upregulation were associated with the proliferation, migration and invasion of lung cancer. Furthermore, the potential functions of miR-192-5p were explored in A549 and NCI-H1299 cells. It was observed that miR-192-5p upregulation suppressed tumour behaviours in lung cancer cells. To further investigate whether miR-192-5p is associated with TRIM44, we used TargetScan software to predict the binding site between miR-192-5p and TRIM44. Luciferase activity assays were performed to verify this prediction. In addition, the significant role of miR-192-5p in negatively regulating TRIM44 expression was manifested by our research group. It was observed that co-transfection with miR-192-5p mimics and TRIM44 reversed some of the effects of TRIM44 on lung cancer cell behaviours.

Ectopic expression of miR-192 may be related to tumour occurrence as it exerts a significant effect on the progression of various malignancies. Recent studies have reported that miR-192 is considerably reduced in many malignant tumours and may be involved in regulating the proliferation, migration, and invasion of cancer cells in neoplasms^20,21,30,31^. For instance, Ji *et al*.^30^ suggested that miR-192-5p was significantly reduced in human bladder cancer. Overexpression of miR-192-5p inhibited the biological function of bladder cancer cells, while downregulation of miR-192-5p contributed to bladder cancer cell proliferation. The authors further demonstrated that miR-192-5p mediated cancer behaviours by targeting YY1. Zhou *et al*.^31^ also reported that miR-192-5p suppressed the initiation and progression of osteosarcoma by directly targeting USP1. In the present study, we observed that overexpression of miR-192-5p suppressed the proliferation, migration and invasion of lung cancer cells.

To elucidate the underlying mechanism of miR-192-5p involved in cell biological function in lung cancer, the TargetScan and miRNA databases were used to identify that miR-192-5p may combine with TRIM44. TRIM44 has been reported to be upregulated in many types of tumours, and TRIM44 is involved in tumour formation and progression^27,32,33^. Accumulating evidence has revealed that TRIM44 enhances the proliferation, migration, and invasion of lung cancer. Luo *et al*.^32^ confirmed that TRIM44 was upregulated in NSCLC tumours and further explored the potential mechanism. It was reported that PDTC can reverse the expression of CXCR6 and MMP9 via TRIM44 to alleviate the promotion of migration and invasion. Xing *et al*.^34^ reported that the dramatic upregulation of TRIM44 is related to early metastasis, high malignancy, poor differentiation, rapid deterioration and poor clinical prognosis. Our results suggest that TRIM44 is a direct target of miR-192-5p. The abundance of miR-192-5p exerted a significant effect on both the mRNA and protein levels of TRIM44. Our observations support that upregulation of miR-192-5p suppresses lung cancer cell proliferation, migration and invasion via targeting TRIM44. The mechanism of the downstream pathway through which miR-192-5p can inhibit lung cancer by targeting TRIM44 should be explored.

In conclusion, our study supports that miR-192-5p was significantly decreased in lung cancer bone metastasis patients and lung cancer cells. Furthermore, our results suggest that miR-192-5p inhibited the proliferation, migration and invasion of lung cancer through TRIM44. Consequently, miR-192-5p may function as a promising therapeutic target for lung cancer bone metastasis; however, further studies are required for implementing lung cancer bone metastasis therapy.

## Methods

### Patients

This study investigated serum from healthy volunteers (n = 130), lung cancer patients without bone metastasis (n = 68) and lung cancer patients with bone metastasis (n = 78). All patients were treated at the Zhengzhou Seventh People’s Hospital (Henan, Chain), and the study was approved by its Institutional Review Boards. The Ethics Committee of Zhengzhou Seventh People’s Hospital approved the procedures. Written informed consent for the use of human materials was approved according to the Zhengzhou Seventh People’s Hospital’s ethical guidelines, and all methods were performed in accordance with the relevant guidelines and regulations.

### Cell culture and transfection

NCI-H1299, H1650, and PC9 cells (cultured in RPMI-1640 medium and 10% foetal bovine serum) were purchased from the Chinese Academy Sciences Cell Bank. A549 cells were obtained from the Laboratory of Immunotherapy of the Affiliated Cancer Hospital of Zhengzhou University (Zhengzhou, China) and maintained in F-12K medium supplemented with 10% FBS. BEAS-2B cells (cultured in LHC basal medium supplement with 10% FBS) were obtained from the Shanghai Cell Biological Institute of the Chinese Academy of Science (Shanghai, China). All cells were maintained in an incubator with a humidified 37 °C atmosphere of 95% air and 5% CO2. miR-192-5p mimic (CUGACCUAUGAAUUGACAGC), miR-192-5p antagomir (CUGACCUAUGAAUUGACAGCC), and negative control miRNA (miR-NC, UUUGUACUACACAAAAGUACUG) were purchased from Thermo Fisher Scientific Company. Amplified miR-192-5p mimic and NC sequences were transfected into the lung cancer cell lines A549 and NCI-H1299 using Lipofectamine 2000 (Invitrogen, USA) according to the manufacturer’s instructions.

### RNA isolation and qRT-PCR amplification

Total RNA extraction and amplification were performed according to the manufacturer’s instructions. The primers were as follows: GAPDH forward 5′-GCACCGTCAAGGCTGAGAAC-3′ and reverse 5′-TGGTGAAGACGCCAGTGGA-3′; miR-192-5p, forward 5′-GGACTTTCTTCATTCACACCG and reverse 5′-GACCACTGAGGTTAGAGCCA; TRIM44 forward 5′-GTGGACATCCAAGAGGCAAT-3′ and reverse 5′-AGCAAGCCTTCATGTGTCCT-3′.

### Luciferase reporter assay

MiR-192-5p mimic, miR-NC, or miR-192-5P antagomir and the pRL-TK vector (Promega Corporation, Madison, WI, USA) carrying the TRIM44 mut or wt 3′UTR were co-transfected into A549 and NCI-H1299 cells by Lipofectamine 2000 (Invitrogen, Carlsbad, CA, USA).

### Western blot analysis

Western blot analysis was performed using an anti-TRIM44 antibody (1:1000; ab220646, Abcam, Cambridge, UK) and rabbit anti-GAPDH antibody (1:1,000; EPR16884, ab181603, Abcam). Images were acquired by scanning with LI-COR’s Odyssey Infrared Imaging System (LI-COR Biotechnology, Lincoln, NE).

### 3-(4,5-dimethylthiazol-2-yl)-2,5-diphenyltetrazolium bromide (MTT) assay

An MTT assay was performed to evaluate the proliferation of lung cancer cells. A549 and NCI-H1299 cells were transfected with miR-192-5p mimic, miR-192-5p antagomir and negative control miRNA using Lipofectamine 2000 (Invitrogen). Each assay was performed in triplicate, and the average absorbance was calculated.

### Migration and invasion assays

Migration and invasion assays using lung cancer cell lines were performed according to the manufacturer’s instructions. After transfection with miR-192-5p mimic, miR-192-5p antagomir and NC miRNA, cells were seeded in the upper chamber of 48-well Transwell® plates (8.0-μm pore size; Costar, Corning Incorporated, Corning, NY). To evaluate the invasion of transfected cells, a polycarbonate membrane (Millipore, Billerica, MA) coated with Matrigel® (Becton Dickinson, Franklin Lakes, NJ) was included.

### Wound-healing assay

To further assess cell invasion, a wound-healing assay was performed in 6-well plates for 24 h. After treated with miR-NC, TRIM44, miR-192-5P + TRIM44 and miR-NC + TRIM44, artificial wound was scratched with a sterile 10-µl pipette tip. Pictures were taken under 100× magnification. The entire experiment was repeated three times.

### Experimental murine models of lung carcinoma bone metastasis

BALB/c-nu/nu mice (aged 6–8 weeks, approximately 30 g) were obtained from Beijing Vital River Laboratory Animal Technology (Beijing, China). This experiment was reviewed and approved by the Institutional Animal Care and Use Committee (IACUC) of Zhengzhou Seventh People’s Hospital, and in all experiments, the mice euthanized according to IACUC guidelines. We made an incision along the right femur of each anaesthetized mouse (chloral hydrate 10%, 0.08-0.12 ml/g). A surgical scalpel was used to drill a tiny hole in the cortex, and A549 cells (5 × 10^6^ cells) were injected into the bone marrow through the hole. Then, the hole was sealed with bone wax, the patellar tendon was reapproximated, and the wound was sutured.

Tumor growth was monitored every 2 days. When tumors reached a size of ~100 mm^3^ (nearly 14 days), mice were randomly divided into three groups (12 mice per group). The first group was treated with miR192-5P antagomir. The second group was treated with miR-NC (non-treated), and the third group was treated with miR192-5Pmimics. These treatments were repeated once after 5 days.

### Immunohistochemistry

Tissue sections were stained by immunohistochemistry to identify TRIM44 protein expression. A primary antibody against TRIM44 (1:200; ab220646, Abcam) was purchased from Abcam.

### Statistical analysis

Differences among the groups were assessed by one-way ANOVA followed by Bonferroni’s multiple comparison test. All data are expressed as the mean ± SEM, and differences of p < 0.05 were considered significant.

## Data Availability

The data used to support the findings of this study are available from the corresponding author upon request.

## References

[1] Lang J, Zhao Q, He Y, Yu X (2018). Bone turnover markers and novel biomarkers in lung cancer bone metastases. Biomarkers: biochemical indicators of exposure, response, and susceptibility to chemicals.

[2] Sarkar D (2017). Statins as Inhibitors of Lung Cancer Bone Metastasis. EBioMedicine.

[3] Zhang L, Gong Z (2017). Clinical Characteristics and Prognostic Factors in Bone Metastases from Lung Cancer. Medical science monitor: international medical journal of experimental and clinical research.

[4] Han S (2019). High CCL7 expression is associated with migration, invasion and bone metastasis of non-small cell lung cancer cells. American journal of translational research.

[5] Wu B (2019). [Comparison of the Survival Time in the Non-small Cell Lung Cancer Patients with Different Organ Metastasis]. Zhongguo fei ai za zhi = Chinese journal of lung cancer.

[6] da Silva GT, Bergmann A, Thuler LCS (2019). Incidence and Risk Factors for Bone Metastasis in Non-Small Cell Lung Cancer. Asian Pacific journal of cancer prevention: APJCP.

[7] Kunikane H (2019). Prospective analysis of the association between skeletal-related events and quality of life in patients with advanced lung cancer (CSP-HOR13). Oncology letters.

[8] Niu Y (2019). Risk factors for bone metastasis in patients with primary lung cancer: A systematic review. Medicine.

[9] Borghetti Paolo, Bonù Marco Lorenzo, Giubbolini Rachele, Levra Niccolo’ Giaj, Mazzola Rosario, Perna Marco, Visani Luca, Meacci Fiammetta, Taraborrelli Maria, Triggiani Luca, Franceschini Davide, Greco Carlo, Bruni Alessio, Magrini Stefano Maria, Scotti Vieri (2019). Concomitant radiotherapy and TKI in metastatic EGFR- or ALK-mutated non-small cell lung cancer: a multicentric analysis on behalf of AIRO lung cancer study group. La radiologia medica.

[10] Park Hyesun, Dahlberg Suzanne E., Lydon Christine A., Araki Tetsuro, Hatabu Hiroto, Rabin Michael S., Johnson Bruce E., Nishino Mizuki (2019). M1b Disease in the 8th Edition of TNM Staging of Lung Cancer: Pattern of Single Extrathoracic Metastasis and Clinical Outcome. The Oncologist.

[11] Chan Jia, Tay Yvonne (2018). Noncoding RNA:RNA Regulatory Networks in Cancer. International Journal of Molecular Sciences.

[12] Weidle UH, Birzele F, Nopora A (2019). MicroRNAs as Potential Targets for Therapeutic Intervention With Metastasis of Non-small Cell Lung Cancer. Cancer genomics & proteomics.

[13] Pradhan Anjan K., Bhoopathi Praveen, Talukdar Sarmistha, Scheunemann Danielle, Sarkar Devanand, Cavenee Webster K., Das Swadesh K., Emdad Luni, Fisher Paul B. (2019). MDA-7/IL-24 regulates the miRNA processing enzyme DICER through downregulation of MITF. Proceedings of the National Academy of Sciences.

[14] Wang H (2019). MiR-183-5p is required for non-small cell lung cancer progression by repressing PTEN. Biomedicine & pharmacotherapy = Biomedecine & pharmacotherapie.

[15] Yan HZ (2019). The expression and clinical significance of miRNA-99a and miRNA-224 in non-small cell lung cancer. European review for medical and pharmacological sciences.

[16] Alcantara KMM, Garcia RL (2019). MicroRNA92a promotes cell proliferation, migration and survival by directly targeting the tumor suppressor gene NF2 in colorectal and lung cancer cells. Oncology reports.

[17] Wang Z, Liu L, Guo X, Guo C, Wang W (2018). microRNA-1236-3p Regulates DDP Resistance in Lung Cancer Cells. Open medicine.

[18] Singh T, Adams BD (2017). The regulatory role of miRNAs on VDR in breast cancer. Transcription.

[19] Hu J (2016). miRNA-223 inhibits epithelial-mesenchymal transition in gastric carcinoma cells via Sp1. International journal of oncology.

[20] Xie X (2019). MiR-192-5p reverses cisplatin resistance by targeting ERCC3 and ERCC4 in SGC7901/DDP cells. Journal of Cancer.

[21] Zhang Y (2019). miRNA-192-5p impacts the sensitivity of breast cancer cells to doxorubicin via targeting peptidylprolyl isomerase A. The Kaohsiung journal of medical sciences.

[22] Jin H, Qiao F, Wang Y, Xu Y, Shang Y (2015). Curcumin inhibits cell proliferation and induces apoptosis of human non-small cell lung cancer cells through the upregulation of miR-192-5p and suppression of PI3K/Akt signaling pathway. Oncology reports.

[23] Ye M (2015). Curcumin promotes apoptosis by activating the p53-miR-192-5p/215-XIAP pathway in non-small cell lung cancer. Cancer letters.

[24] Wang H, Fang ZL, Zhang GH, Ma X (2018). TRIM44, a crucial target of miR-410, functions as a potential oncogene in osteosarcoma. OncoTargets and therapy.

[25] Kawabata Hidetaka, Azuma Kotaro, Ikeda Kazuhiro, Sugitani Ikuko, Kinowaki Keiichi, Fujii Takeshi, Osaki Akihiko, Saeki Toshiaki, Horie-Inoue Kuniko, Inoue Satoshi (2017). TRIM44 Is a Poor Prognostic Factor for Breast Cancer Patients as a Modulator of NF-κB Signaling. International Journal of Molecular Sciences.

[26] Yamada Y (2017). A novel prognostic factor TRIM44 promotes cell proliferation and migration, and inhibits apoptosis in testicular germ cell tumor. Cancer science.

[27] Tan Y, Yao H, Hu J, Liu L (2017). Knockdown of TRIM44 Inhibits the Proliferation and Invasion in Prostate Cancer Cells. Oncology research.

[28] Kashimoto K (2012). Overexpression of TRIM44 contributes to malignant outcome in gastric carcinoma. Cancer science.

[29] He J (2019). MicroRNA-20b promotes proliferation of H22 hepatocellular carcinoma cells by targeting PTEN. Oncology letters.

[30] Ji D, Jiang L, Li Y (2018). MiR-192-5p suppresses the growth of bladder cancer cells via targeting Yin Yang 1. Human cell.

[31] Zhou S (2018). MicroRNA-192-5p suppresses the initiation and progression of osteosarcoma by targeting USP1. Oncology letters.

[32] Luo Q (2015). Trim44 facilitates the migration and invasion of human lung cancer cells via the NF-kappaB signaling pathway. International journal of clinical oncology.

[33] Liu S, Yin H, Ji H, Zhu J, Ma R (2018). Overexpression of TRIM44 is an independent marker for predicting poor prognosis in epithelial ovarian cancer. Experimental and therapeutic medicine.

[34] Xing Y (2016). TRIM44 promotes proliferation and metastasis in nonsmall cell lung cancer via mTOR signaling pathway. Oncotarget.

